# Supplementary material for: The discovery of novel noncoding RNAs in 50 bacterial genomes
Source: Nucleic Acids Res. 2024 Apr 22;52(9):5152–65. doi: 10.1093/nar/gkae248 (PMC11109978; doi:10.1093/nar/gkae248)
Supplement: gkae248_Supplemental_Files [file gkae248_supplemental_files.zip › Narunsky 50 Genomes RNA Motifs Files.zip!/Publication/69-E_rhusiopathiae/Figures/LRC-69-4-NC_015601_1_IGR_21_1463459_1463839.sto.pdf]

LRC-69-4-NC_015601_1_IGR_21_1463459_1463839.sto.r2r

5′-UCUUCGACAAAAACA●YUAAAA●C●A●CA●●Y●●UAACACUG●●YR●●R●U●UCGACACU●○●UU●UCR●YAAUAR●Y●AU●UYA●RR●RA●ACUGUCGA●AY●U●●RUY●AUA●YC●UAGAUUA●CACA●R●YYA●U●CRA●●CAYYCARUUCUC●●AAU●U○○○○U●AU●UC●●C●U●UAYY●YYYAUCACAUAAA●ACACACAUUU●UUUCACUUUUUAUUGACAGCGGUUACA●C●CRUGCUAAUAUGUGCUUGUAAGAUAGGAUUGUUACUGAAGAAGGCAAGACCUACAAAUAUGGC●●UACGCUAUUUUUGUAGGUCUUUUUAYRUCUUY○○G●ARYAAUUYCU●AAUCAUAUAGAAAGGAGAGUGA●UU○
